# The interaction between pain and mental disorders: An interdisciplinary analysis through bibliometrics

**DOI:** 10.1002/ibra.70003

**Published:** 2025-09-29

**Authors:** Zhimin Tan, Qiyu He, Xian Zhang, Jiarong Feng, Yuxin He, Xiaoqiang Li

**Affiliations:** ^1^ Department of Anaesthesiology West China Hospital of Sichuan University Chengdu China; ^2^ Department of Urology West China Hospital of Sichuan University Chengdu China; ^3^ Khoury College of Computer Sciences Northeastern University Boston Massachusetts USA; ^4^ Department of Gastroenterology and Hepatology The First Affiliated Hospital of Soochow University Suzhou China

**Keywords:** bibliometrics, mental illness, neuroimmune interactions, pain

## Abstract

The evolving research on the interactions between pain and mental disorders underscores the critical role of neuroimmune interplay in shaping pain perception and mental illness progression. This study employs bibliometric analysis to scrutinize the research landscape, identify emerging hotspots, and forecast future directions. A systematic review of literature from 2014 to 2023 was conducted using CiteSpace software for co‐citation analysis, keyword co‐occurrence, and burst detection to identify research hotspots and trends. The study examined developmental trends in pain and psychiatric disorder research, highlighting major research institutions and key themes. It unveils pivotal contributors and collaborative networks, showing significant growth in recent years. Emphasis is placed on neuroinflammation and neuroimmunomodulation interactions with mental illnesses. Keyword and thematic clustering analyses highlight the roles of microglial activation, inflammatory mediators, neurotransmitters, and emotional regulation processes. This study paves the way for future inquiries into neuroimmune mechanisms, the development of personalized treatment strategies, and an interdisciplinary approach to enrich our understanding of the biopsychosocial model in these conditions. Future studies should delve deeper into the molecular intricacies of these interactions to develop more effective therapeutic strategies, aiming to enhance patients' quality of life.

## Graphical abstract

This study elucidates the complex relationship between pain and mental disorders via bibliometric analysis. The left section illustrates neural pathways of pain, while the upper right highlights the link between pain and mental states like anxiety, stress, and sleep disorders, showing their interaction in exacerbating pain perception and persistence. The lower right flowchart outlines the bibliometric methodology, including a global research overview, literature analysis, and detailed exploration of pain mechanisms. Through co‐occurrence, cluster, and co‐citation analyses, this study examines the connections between pain, neuroinflammation, oxidative stress, and mental health, emphasizing the roles of peripheral perception and central sensitization in pain transmission. It also reveals significant mechanisms such as neuropathic pain and cognitive impairment. This study aims to guide future studies and offer new perspectives for personalized treatment strategies.

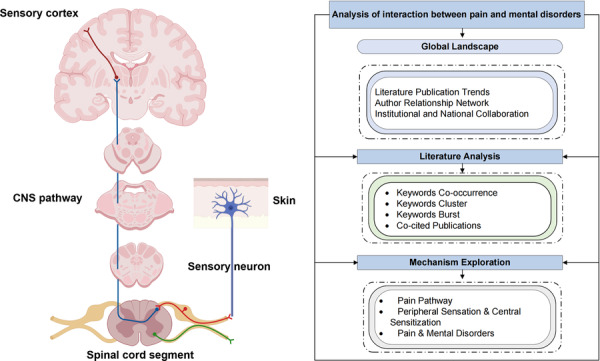

## 1 INTRODUCTION

Pain constitutes a multifaceted sensory and emotional phenomenon characterized by intricate interactions among the nervous and immune systems.^1^ Mental disorders such as depression, anxiety, and schizophrenia exert influence on the neuroimmune network, potentially exacerbating or ameliorating pain perception. With advancing research, there is growing recognition of pain as a phenomenon shaped by multisystem interactions, with significant collaboration between the nervous, immune, and endocrine systems.^2^ Within psychiatric disorders, this interplay assumes heightened complexity, impacting the perception and modulation of pain, and participating in the modulation of neuroinflammatory processes.

As research delves deeper into the intricate interplay between pain and psychiatric disorders, it not only enhances our comprehension of pain mechanisms but also offers fresh perspectives for the development of therapeutic interventions. For example, mental disorders impact pain perception by engaging the neuroendocrine‐immune network, where neuroimmune interactions play a crucial role in modulating pain triggered by psychiatric conditions.^3^, ^4^, ^5^ Immune cells like microglia and T cells are involved in the initiation and perpetuation of pain by releasing inflammatory mediators.^6^

While significant strides have been made in elucidating the intricate interplay among pain, mental illness, neuroimmunity, and neuroinflammation, numerous challenges persist. The intricacy of pain mechanisms involves a wide array of cellular types and molecular pathways, compounded by individual variabilities in pain and psychiatric system responses, rendering the discovery of universal therapeutic approaches particularly challenging.^7^, ^8^ Future research demands deeper exploration of the biological underpinnings of these variances and their integration into personalized pain management strategies. Furthermore, research into key molecules and signaling pathways involved in neuroimmune interactions will be imperative. The adoption of interdisciplinary approaches and the fostering of close collaboration among disciplines such as basic science, clinical medicine, psychology, pharmacy, and others will be pivotal in addressing this intricate issue.

Bibliometric analysis empowers researchers to efficiently analyze and visualize extensive datasets, discern trends and core issues, and delve into novel research avenues,^9^ such as investigating how specific immune cells modulate pain responses in aberrant mental states or devising innovative therapeutic strategies. This methodology not only facilitates the identification of pivotal areas but also propels interdisciplinary research progress, enhancing our comprehension of pertinent mechanisms and furnishing a scientific framework for subsequent exploration.

This study employs bibliometric analysis to systematically review the current state and development trends in research on the interplay between pain, mental disorders, and neuroimmune interactions. The objective is to identify core research themes and hotspots, assess the influence of leading scholars and their contributions, analyze interdisciplinary collaboration networks, and highlight key literature and high‐impact studies. Additionally, the study aims to project future directions in personalized pain management and multidisciplinary integration, providing a scientific basis and reference for improving diagnostic and therapeutic approaches.

## 2 MATERIALS AND METHODS

### 2.1 Literature search

In this study, a comprehensive literature search was conducted across several prominent academic databases, including PubMed, Web of Science, and Scopus. The search strategy was carefully designed to include keywords directly related to the research topic: Title/Abstract = (pain) AND [(aberrant mental states) OR (mental system) OR (psychiatric disorders) OR (neuroimmunity) OR (neuroinflammation)]. Two authors independently performed the search on the same day, covering the period from January 1, 2014, to December 1, 2023.

### 2.2 Inclusion and exclusion criteria

The inclusion criteria for this study were peer‐reviewed original research articles and review papers on pain and mental disorders, sourced from PubMed, Web of Science, and Scopus, and published in English. Exclusion criteria included articles that were not formally published, non‐English publications, articles published more than a decade ago, duplicates, and certain types of publications such as letters, conference abstracts, book chapter, and editorials. Additionally, articles not directly related to pain and mental disorders were excluded.

**Figure 1:**
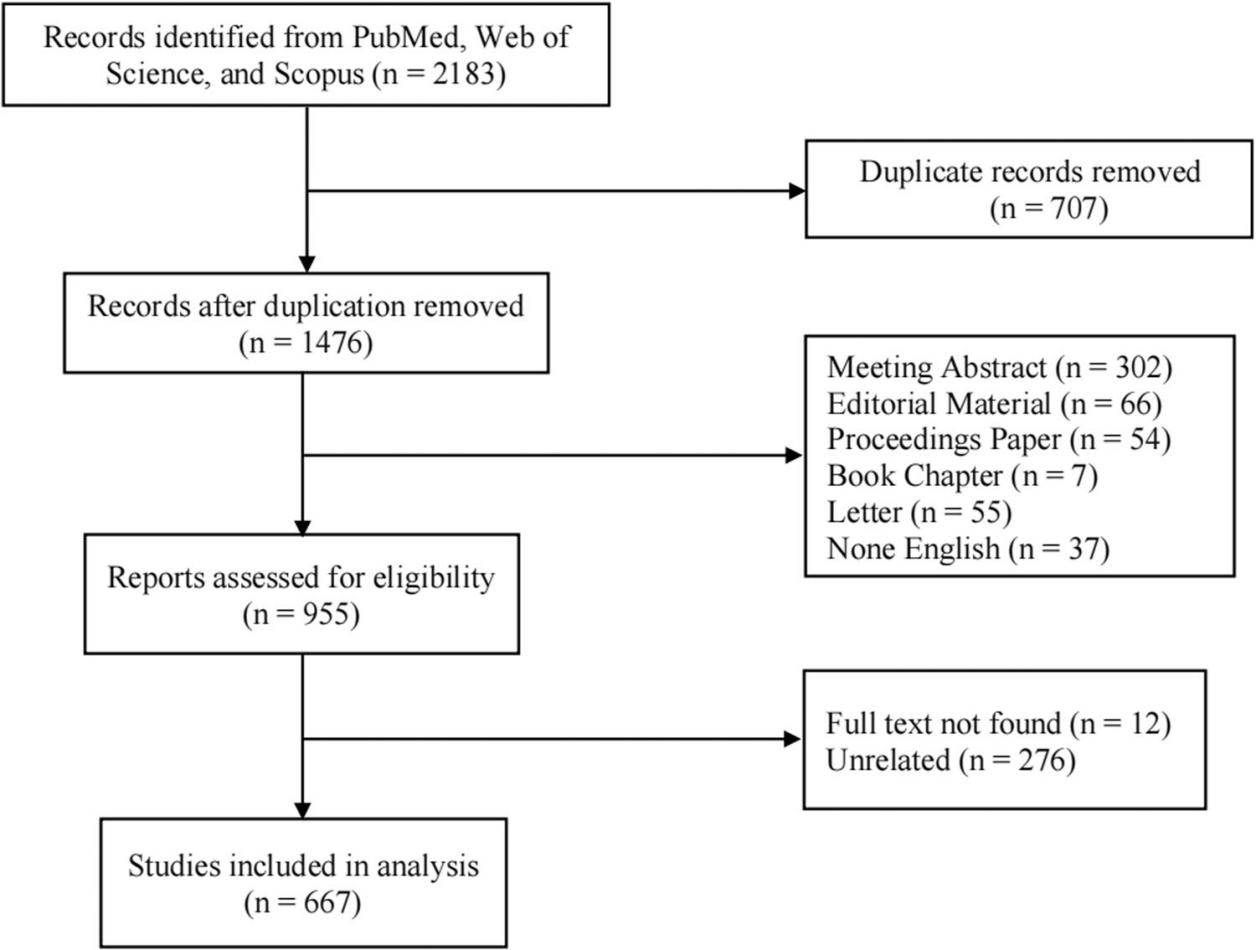
The flowchart of literature inclusion and exclusion.

Two reviewers independently assessed the literature. The initial screening was based on the titles and abstracts of the articles. A secondary screening was then conducted according to the inclusion and exclusion criteria. Any discrepancies during the screening process were resolved by a third reviewer, who read the full text of the disputed articles. The reviewers discussed and reached a consensus on whether to include or exclude the articles, resulting in the inclusion of 667 records (Figure 1).

### 2.3 Literature data extraction

The search results were downloaded and exported to CiteSpace software for further analysis. The data were saved in Plain Text format, with Full Record and Cited References selected as the record content. This export included key information for each article, such as author names, affiliations, countries, publication years, journal titles, article titles, abstracts, keywords, and references. Additionally, manual correction and normalization were applied to address variations in the expression of author names, institution names, and keywords.

### 2.4 Data analysis and presentation of results

In‐depth data analysis was conducted using CiteSpace software, following the principles of bibliometric analysis.^10^ We performed co‐occurrence analyses of countries, authors, institutions, and co‐cited references to generate visual atlases. CiteSpace also facilitated cluster and burst analysis of keywords, with cluster analysis using unsupervised learning to model the structure of knowledge based on annual publication trends.^10^ The resulting network diagrams illustrate the frequency of co‐occurrences (node size) and their relationships (connections).^11^ Notably, nodes with a purple outer ring indicate high centrality (>0.1), suggesting significant potential for scientific influence.^12^ Our analysis also included burst detection to identify emerging research hotspots and topics. The results are presented through network diagrams, trend charts, and quantitative analyses, providing a comprehensive overview of the field's research landscape.

## 3 RESULT

### 3.1 Analysis of literature publication trends

**Figure 2:**
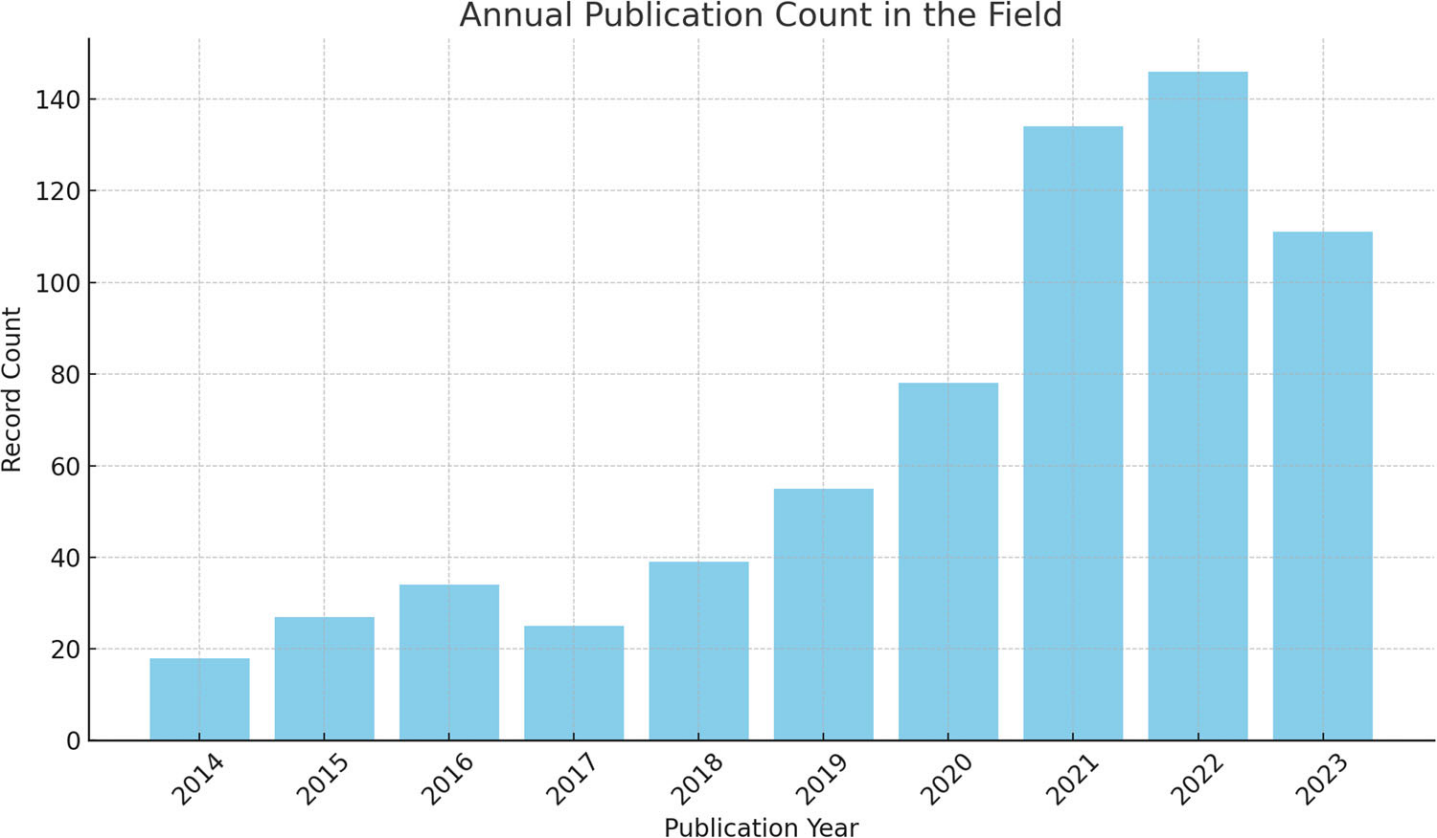
Annual publication trend in the field of pain and mental illness. [Color figure can be viewed at wileyonlinelibrary.com]

From 2014 to 2023, the annual number of publications in the field of pain and mental disorders exhibited significant fluctuations (Figure 2). The highest publication count occurred in 2022, with 146 publications, accounting for approximately 21.9% of the total, followed by 2021 with 134 publications (20.1%). Although data for 2023 is not yet fully compiled, 111 publications have already been recorded, representing 16.6% of the total. This trend indicates a growing research interest in this field, particularly in recent years, likely due to increased recognition of the complex interactions between pain and mental disorders. The sharp rise in publications during 2021 and 2022 may reflect advancements in research methodologies, technological innovations, and a heightened focus on understanding the biological underpinnings and clinical significance of these interactions.

### 3.2 Analysis of author relationship network

**Figure 3:**
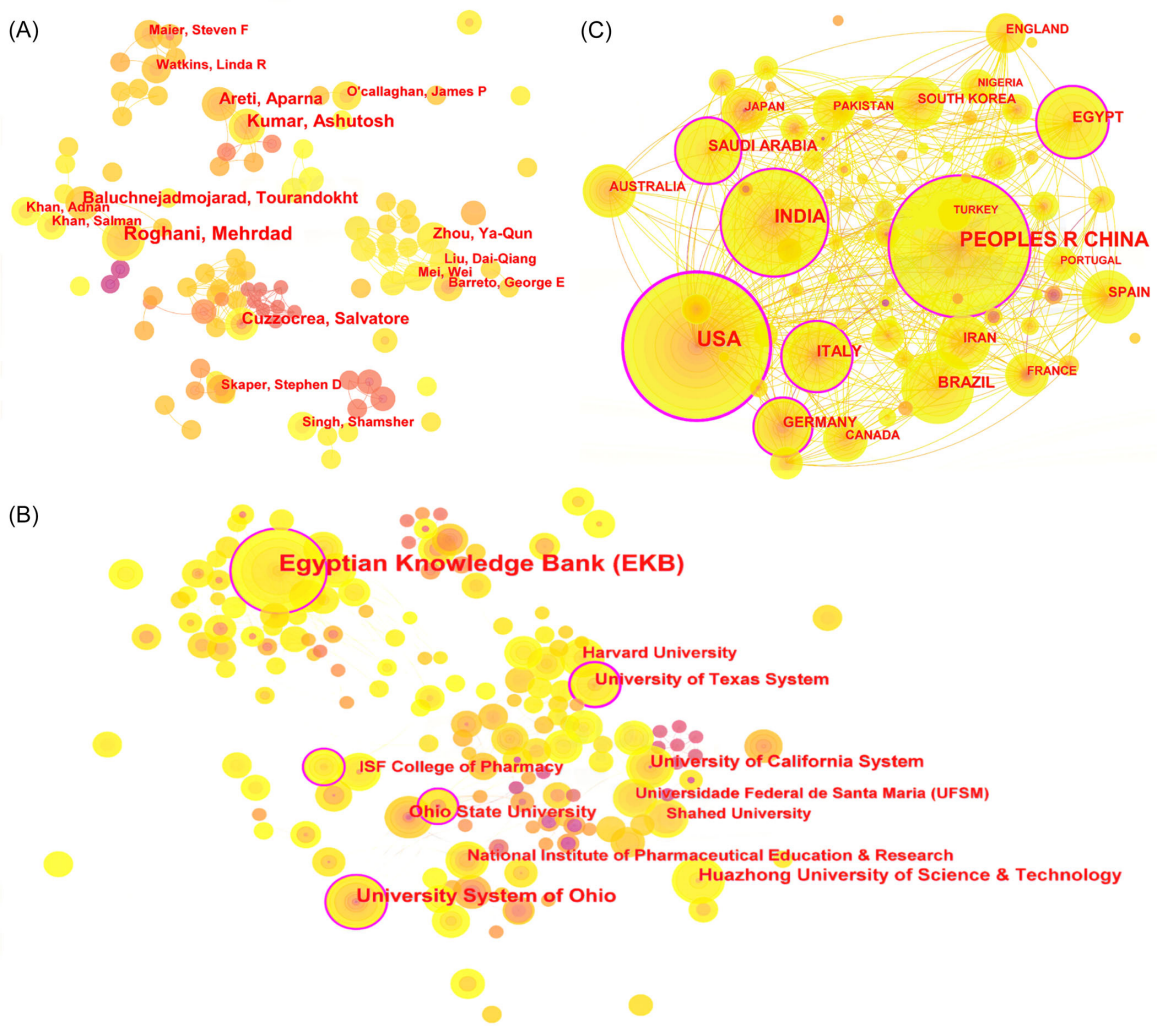
Collaboration network in mental illness and pain research (2014–2023). (A) Author collaboration network in mental illness and pain research. (B) Institutional collaboration network in mental illness and pain research. (C) National collaboration network in mental illness and pain research. [Color figure can be viewed at wileyonlinelibrary.com]

As shown in Figure 3A, collaboration among authors over the past decade reveals a highly interconnected network of researchers. Key contributors such as Baluchnejadmojarad, Tourandokht, and Roghani, Mehrdad stand out due to their central positions and larger node sizes, indicating high publication frequency and significant impact in the field. The research conducted by Roghani and his team focuses on the role of neuroinflammation in diseases such as epilepsy, autism, and Alzheimer's, providing crucial insights into how inflammatory processes contribute to neurological and psychiatric disorders. Their work emphasizes the importance of targeting neuroinflammatory pathways as potential therapeutic strategies.^13^, ^14^, ^15^ Similarly, Areti's team and Kumar's team have made significant contributions by elucidating the roles of oxidative stress and mitochondrial dysfunction in chronic pain.^16^, ^17^ These collaborations underscore the interdisciplinary nature of this study, where insights from neuroscience, molecular biology, and clinical medicine converge to enhance our understanding of the complex interplay between pain and mental health.

### 3.3 Institutional and national collaboration analysis

Figure 3B illustrates the pivotal roles of institutions such as the Egyptian Knowledge Bank (EKB), the University of Texas System, and the University System of Ohio in advancing research within this field. These institutions, characterized by extensive publication records and high centrality indices, are instrumental in shaping the research landscape. The United States, with 211 publications and a centrality index of 0.46, leads the field, reflecting its robust research infrastructure and substantial funding. Chinese institutions also exhibit significant influence, with 179 publications and a centrality index of 0.13, highlighting their expanding role in global research (Figure 3C and Table 1).

**Table 1:** Institutional/National collaboration network in mental illness and pain research (2014–2023).

Freq	Centrality	Country	First publication year	Freq	Centrality	Institution	First publication year
211	0.46	USA	2000	44	0.14	Egyptian Knowledge Bank (EKB)	2012
52	0.14	ITALY	2008	20	0.1	University System of Ohio	2002
41	0.13	SAUDI ARABIA	2012	14	0	Huazhong University of Science & Technology	2016
29	0.09	SPAIN	2005	13	0.05	University of California System	2004
45	0.17	EGYPT	2012	13	0	Ohio State University	2002
179	0.13	PEOPLES R CHINA	2010	12	0.23	University of Texas System	2016
112	0.11	INDIA	2008	10	0.03	Harvard University	2019
18	0.09	FRANCE	2004	10	0.07	National Institute of Pharmaceutical Education & Research	2013
31	0.06	IRAN	2013	10	0	ISF College of Pharmacy	2011
38	0.12	GERMANY	2002	9	0.01	Universidade Federal de Santa Maria (UFSM)	2019

Institutions in developed countries benefit from resource advantages and central positions, while those in developing countries demonstrate considerable potential through strategic investments in education and research. These findings suggest that nations with established research infrastructures and strong institutional support are at the forefront of advancing our understanding of pain and mental disorders. Network analysis further underscores the importance of cross‐institutional collaboration in fostering innovation and addressing the multifaceted challenges of these conditions, particularly as research increasingly integrates neuroscience, psychology, and clinical medicine.

### 3.4 Co‐occurrence and cluster analysis

**Figure 4:**
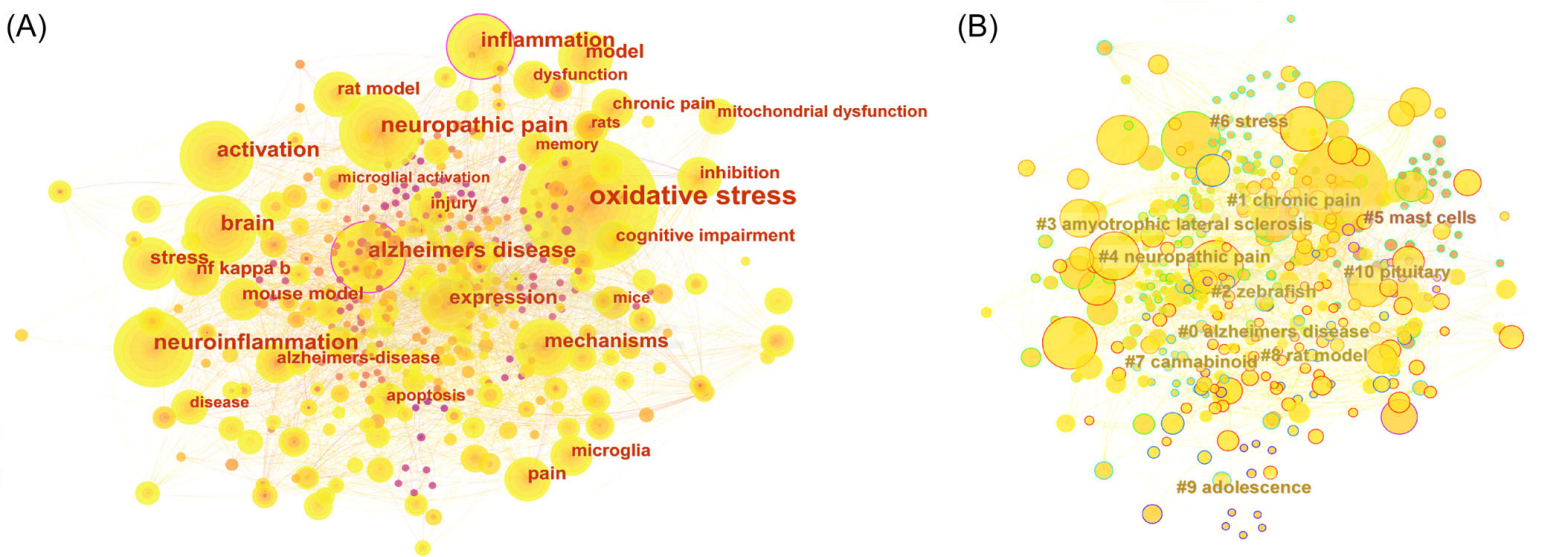
Network diagram keywords analysis. (A) Keyword co‐occurrence analysis. (B) Keyword clustering analysis. [Color figure can be viewed at wileyonlinelibrary.com]

The co‐occurrence network analysis (Figure 4A) elucidates the logical connections and underlying mechanisms among keywords in the field of pain and mental disorders. For example, the strong associations of Alzheimer's disease with neuroinflammation, oxidative stress, neuropathic pain, and cognitive impairment highlight the pivotal roles of neuroinflammation and oxidative stress in the pathogenesis of neurodegenerative diseases and pain mechanisms. These pathological processes jointly impact the brain, leading to cognitive impairment, a hallmark of Alzheimer's disease and intricately linked with chronic pain syndromes.^18^ The connections between pain and inflammation and between neuropathic pain and microglia emphasize the critical role of immune responses in the onset and maintenance of pain, particularly neuropathic pain, where microglial activation is paramount. Activated microglia release pro‐inflammatory cytokines such as interleukin‐1β (IL‐1β), tumor necrosis factor‐α (TNF‐α), and interleukin‐6 (IL‐6), which sensitize pain pathways and exacerbate pain perception, underscoring the complex interplay between the immune system and pain pathways.^19^

Furthermore, the relationship between oxidative stress and mitochondrial dysfunction reveals common pathological processes in various neurodegenerative diseases and pain models. Oxidative stress induces mitochondrial dysfunction, a significant factor in the development of neurodegenerative changes and chronic pain. Mitochondrial dysfunction, in turn, intensifies oxidative stress, creating a vicious cycle that exacerbates neuronal damage and disease progression. This interaction not only contributes to cognitive decline but is also closely related to the onset of pain syndromes.^20^, ^21^

The keyword cluster analysis, using co‐occurrence frequency and clustering algorithms, identified distinct research themes (Figure 4B). The Alzheimer's Disease cluster (#0) examines the link between cognitive decline and chronic pain, and the impact on mental states such as depression and anxiety. This suggests that mental health issues may be caused by neurodegenerative diseases. The Chronic Pain cluster (#1) investigates the long‐term impact of persistent pain on quality of life, particularly the bidirectional relationship between chronic pain and co‐existing mental disorders like depression and anxiety, where symptoms exacerbate each other. Management strategies within this cluster emphasize integrated care, including psychological support and interventions.

The Amyotrophic Lateral Sclerosis (ALS) cluster (#3) focuses on pain management in ALS patients, exploring how neurodegenerative processes cause muscle pain and mental health issues such as anxiety and depression. The most common reported locations of pain in patients with ALS are the back (50%), followed by the limbs (47%) and joints (42%). Notably, insomnia has been linked to an increased predisposition to facial, abdominal, and knee pain, while anxiety has been associated with a higher likelihood of neck/shoulder and back pain.^22^ These studies investigate treatment strategies to alleviate both physical and psychological distress, underscoring the need for comprehensive care for ALS patients. Additionally, the cluster analysis reveals the Neuropathic Pain theme (#4), exploring pain resulting from nerve damage, its relief strategies, and the interaction between pain and the nervous system.

Other themes, such as Mast Cells (#5), Cannabinoids (#7), and Rat Models (#8), highlight the importance of experimental applications. Mast cells, as biomarkers, are associated with the inflammatory response in pain, exacerbating nociceptive sensitivity in conditions such as fibromyalgia and irritable bowel syndrome.^23^, ^24^ The endogenous cannabinoid system plays a key role in modulating pain and the stress response in fibromyalgia. Clinical studies have identified alterations in this system among fibromyalgia patients, including single‐nucleotide polymorphisms and elevated circulating levels of endocannabinoids and related N‐acylethanolamines. Preclinical evidence suggests that inhibition of fatty acid amide hydrolase can alleviate pain‐ and anxiety‐related behaviors.^25^

### 3.5 Keyword burst analysis

**Figure 5:**
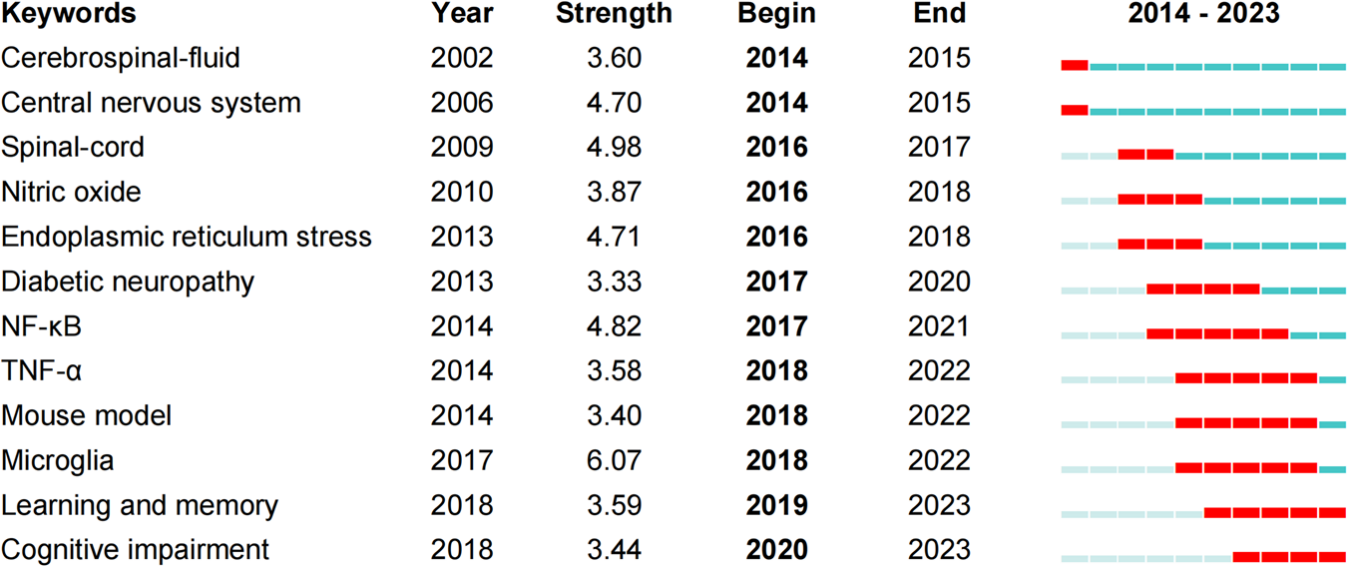
Top 12 Keywords with the strongest citation bursts. [Color figure can be viewed at wileyonlinelibrary.com]

The keyword burst analysis (Figure 5) reveals the timeline of research progress and evolving focus areas in the field of Pain and Mental Disorders. Since 2014, the citation intensity and time window of various keywords have shifted, reflecting changes in research interest and scientific exploration. Initially, the focus was on keywords such as central nervous system, spinal cord, and cerebrospinal fluid, highlighting a strong interest in the fundamental structures and functions of the nervous system. From 2016 to 2020, keywords like nitric oxide, diabetic neuropathy, nuclear factor‐ kappaB (NF‐κB), and endoplasmic reticulum stress emerged as hot topics, indicating a growing demand for understanding the molecular mechanisms underlying pain and mental disorders. NF‐κB, in particular, has garnered significant attention due to its critical role in regulating inflammation and immune responses, as well as its close association with pain and various mental disorders.

In recent years, the increased citation frequency of keywords such as microglia, learning and memory, and cognitive impairment indicates a shift in research focus towards understanding behavioral and functional disorders within the nervous system, particularly concerning the interplay between pain and mental disorders. Microglia, the primary immune cells of the brain, have garnered significant attention for their roles in neurodegenerative diseases and psychiatric disorders. Studies have shown that nerve injury, inflammation, and the accumulation of pain‐related substances alter the spinal microenvironment, activating microglia and astrocytes.

Damaged neurons release chemokines such as C–C motif chemokine ligand 2 (CCL2), CCL21, C‐X‐C motif chemokine ligand 10 (CXCL10), and Fractalkine, which activate microglia through receptors like C–C chemokine receptor 2 (CCR2), C‐X‐C chemokine receptor 3 (CXCR3), and C‐X3‐C motif chemokine receptor 1 (CX3CR1). Activated glial cells further trigger MAPK signaling pathways (including p38, ERK, and JNK), leading to the nuclear translocation of NF‐κB and the release of inflammatory mediators such as TNF‐α, IL‐1β, IL‐6, and prostaglandins. These inflammatory mediators not only activate primary afferent neurons and dorsal horn neurons but also sensitize nociceptors and modulate excitatory synaptic transmission in the central nervous system.^26^, ^27^, ^28^ This process underscores the complex roles of immune responses and inflammatory mediators in the nervous system's pain perception and the development of psychiatric disorders, making it a current hotspot and frontier area of research.

### 3.6 Analysis of co‐cited literature

**Figure 6:**
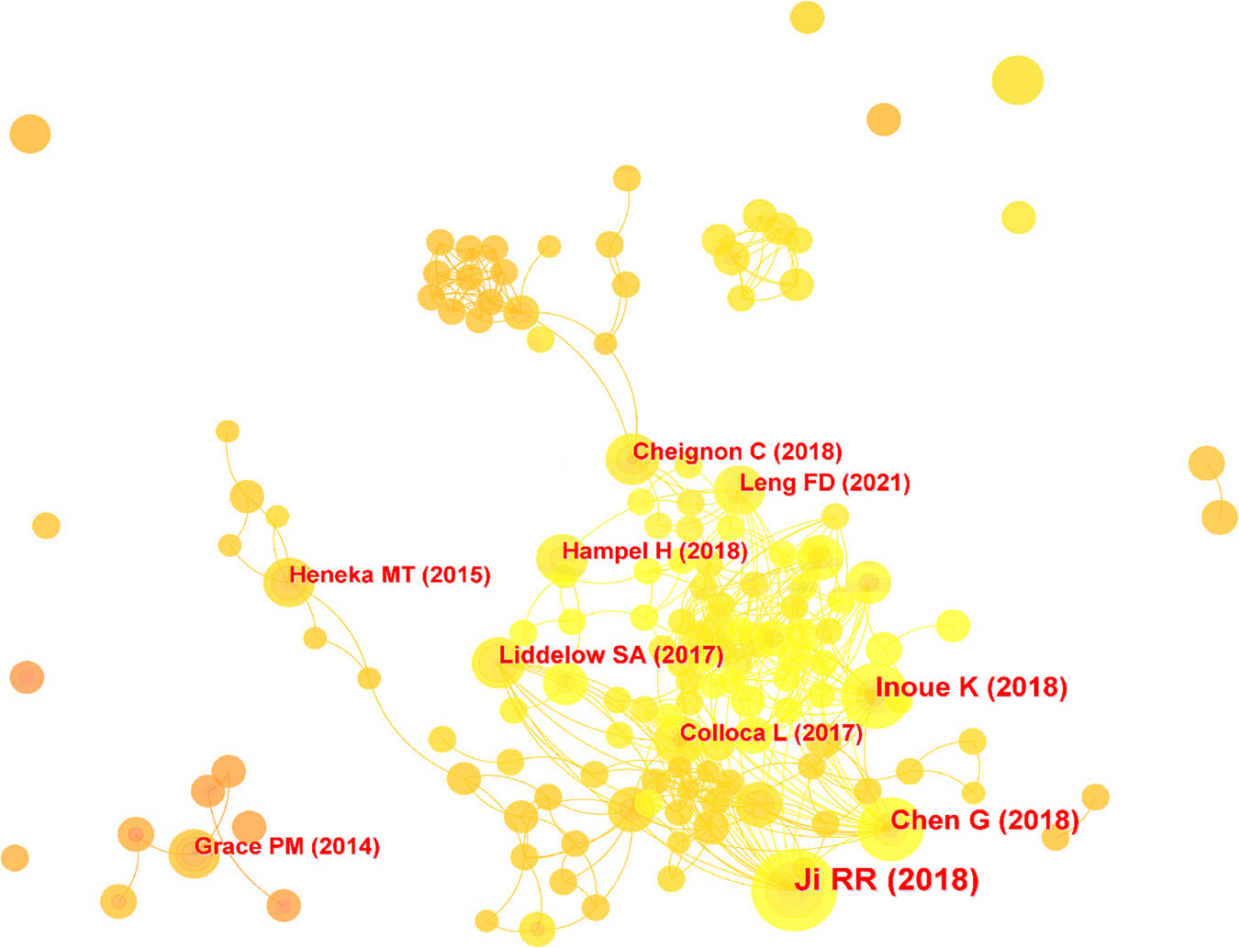
Co‐citation analysis in pain and mental health research (2014–2023). [Color figure can be viewed at wileyonlinelibrary.com]

We performed a co‐citation analysis of the literature in the field of pain and mental disorders (Figure 6), with the top 10 most frequently cited articles summarized in Table 2. These seminal papers are published in high‐impact journals such as *Nature*, *Nature Reviews Immunology*, and *Nature Reviews Neuroscience*, which hold significant academic influence in neuroscience, immunology, and biochemistry. Additionally, journals like *Anesthesiology*, *Neuron*, and *Redox Biology* are more focused on specific areas such as pain research, neuronal biology, and oxidative stress. These highly cited studies delve into key mechanisms such as neuroinflammation, microglial activation, amyloidosis, and tau protein pathology, highlighting their significance in the pathogenesis and treatment of pain and mental disorders. The most cited paper provides a detailed analysis of the persistence of chronic pain within the central nervous system, emphasizing the role of neuroinflammation in this process.^29^ It also explores the mechanisms by which cytokines and chemokines amplify pain and examines the influence of sex differences on pain pathways, offering new perspectives for personalized treatment strategies.

**Table 2:** Top 10 highly co‐cited literature in pain and mental disorders field (2014–2023).

Literature	Co‐citation frequency	Blast strength	Centrality	Publication journal
Ji RR (2018)^29^	29	4.54	0.02	*Anesthesiology*
Chen G (2018)^30^	17	0	0.06	Neuron
Inoue K (2018)^31^	16	0	0.06	*Nat Rev Neurosci*
Liddelow SA (2017)^32^	12	3.5	0.01	*Nature*
Cheignon C (2018)^33^	12	2.49	0.06	*Redox Biol*
Hampel H (2018)^34^	10	3.99	0	*Brain*
Grace PM (2014)^35^	10	3.81	0.04	*Nat Rev Immunol*
Heneka MT (2015)^36^	10	3.72	0.02	*Lancet Neurol*
Colloca L (2017)^37^	10	0	0	*Nat Rev Dis Primers*
Leng FD (2021)^18^	10	0	0.01	*Nat Rev Neurol*

**Figure 7:**
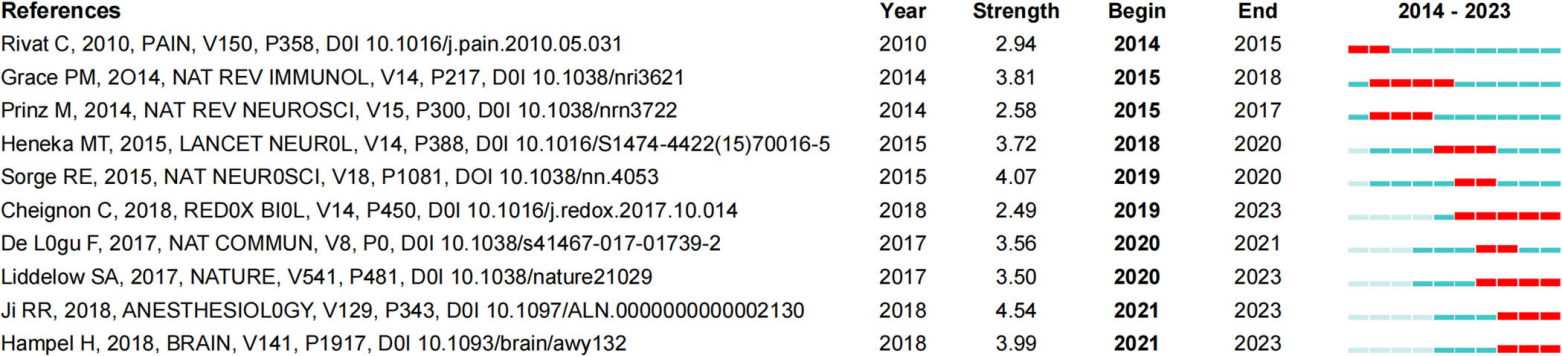
Top 10 literatures with the strongest citation bursts in pain and mental health (2014–2023). [Color figure can be viewed at wileyonlinelibrary.com]

The burst analysis of co‐cited literature (Figure 7) demonstrates a marked increase in citation frequency in recent years, underscoring their central role in driving the field forward. Early studies (before 2015) focused primarily on the relationship between immune responses in the nervous system and pain mechanisms, with particular attention to the effects of psychological stress and neuroimmune diseases on neural function. As research evolved, attention shifted to the interaction between neurodegenerative diseases and immune mechanisms, specifically the connections among Alzheimer's disease, neuropathic pain, and chronic pain. In recent years (post‐2020), research has further concentrated on the intricate mechanisms of neurodegenerative diseases, pain perception, and neuroinflammation. These studies highlight the critical roles of these mechanisms within the central nervous system and explore how they interact to influence the clinical manifestations of diseases. Such findings provide a theoretical foundation for developing novel therapeutic strategies and lay the groundwork for future research into these complex mechanisms and their roles in disease progression.

## 4 DISCUSSION

### 4.1 What have we discovered?

The bibliometric analysis aimed to uncover evolutionary trends, hot topics, and future frontiers in pain and mental illness research. Recent years have witnessed a sustained growth in this field, reflecting its increasing significance and attention. Interdisciplinary collaborations have surged, amalgamating insights from neuroscience, psychology, and clinical medicine, thus enriching research methodologies and perspectives. Analysis reveals a decade‐long emphasis on neurodegenerative and painful diseases, pain generation mechanisms, and the interface between pain and mental illness. Future research is poised to adopt the biopsychosocial model, offering comprehensive insights into their intricate relationship. Innovative methodologies like neuroimaging and molecular biology, alongside intensified interdisciplinary collaborations, will drive further progress, paving the way for personalized treatments and precision medicine.

### 4.2 Global landscape of research on pain and mental system disorders

In the realm of research on pain and mental illness, research institutions worldwide exhibit varying publication volumes and centrality indices, reflecting the status and influence of countries and institutions within the global research network. The United States leads with 211 papers and a centrality index of 0.46, attributed to its mature infrastructure and funding support. Developing countries such as China, India, Egypt, and Saudi Arabia, though having fewer papers than the United States, demonstrate strong global influence as indicated by their centrality. European countries like Italy, Spain, and Germany also play significant roles in research on pain and mental illness, showcasing Europe's solid foundation in this field. Overall, institutions in developed countries hold resource advantages and central positions, while those in developing countries exhibit significant potential through education and research investment.

Over the past decade, research on pain and mental illness has undergone significant development and transformation. Studies have intricately explored pain mechanisms, highlighting its complex interactions with the nervous, endocrine, and oxidative stress systems. This scientific exploration has not only deepened our understanding of pain's biological underpinnings but also led to the emergence of novel treatment strategies, including biologics, antioxidants, neuroregulation techniques, and psychological interventions. Moreover, the advent of personalized medicine has revolutionized pain management, offering tailored and more effective treatment options through bioinformatics‐driven identification of individual variations. These advancements not only enhance patients' quality of life but also open up new avenues for future pain research and treatment approaches.

### 4.3 Pain perception: From peripheral sensory activation to central sensitization

Pain is a complex response to noxious stimuli, involving various biological processes and neural pathways (Table 3). It begins with the activation of nociceptors—specialized receptors in peripheral tissues—that convert stimuli into electrical signals via specific ion channels like sodium and calcium channels. These signals then travel through primary afferent neurons to the central nervous system, where neurotransmitters and neuropeptides are released in the spinal cord's dorsal horn for initial processing. Secondary neurons further transmit the signals to the brain, where the location, intensity, and nature of pain are finely decoded.^35^ The brain modulates pain processing through descending pain modulation systems, employing serotonergic and noradrenergic pathways to regulate pain perception.^38^ Internally, two main brain pathways encode the sensory‐discriminative and affective‐cognitive aspects of pain, integrating emotional responses and cognitive evaluations to shape the complex pain experience.^39^

**Table 3:** The process of pain generation.

Stage	Descriptinon
Peripheral Activation	Injury or stimuli activate nociceptors, converting stimuli into electrical activity.
Central Transmission	Electrical signals are transmitted via primary afferent neurons to the central nervous system (CNS), reaching the spinal cord.
Neurotransmitter Release	At the spinal cord, neurotransmitters like glutamate and neuropeptides facilitate signal transmission to secondary neurons.
Central Processing	Secondary neurons project to higher brain centers (e.g., thalamus, somatosensory cortex) for pain localization, intensity, and quality encoding.
Descending Modulation	Brain regions regulate pain signal transmission at the spinal level through descending pathways, involving serotoninergic and noradrenergic neurons.
Emotional and Cognitive Integration	Brain areas like the prefrontal cortex and limbic system integrate emotional and cognitive aspects, affecting pain perception.

Pain sensitization is a complex process involving intricate coordination between the nervous and immune systems (Table 4). When pain sensitivity is heightened, innocuous stimuli may induce pain or exacerbate and prolong responses to noxious stimuli.^40^

**Table 4:** The key systems in the pain sensitization process.

Component	Process involved	Key participants	Mechanism of action	Pain sensitization
Peripheral Nervous System	Peripheral Activation	Nociceptors, Peripheral Nerve Fibers	Nociceptors respond to stimuli under injury and inflammation, transmitting pain signals to the central nervous system through nerve fibers.	Peripheral sensitization reduces the response threshold of nociceptors to stimuli, making normally non‐painful stimuli cause pain.
Immune System	Peripheral Immune Response	TNF‐α, IL‐1β, Immune Cells	Noxious stimuli cause peripheral inflammation, immune cells release inflammatory mediators, promoting sensitization of nerve endings.	Inflammatory mediators enhance the excitability of nerve fibers, promoting amplification and persistence of pain signals.
	Central Immune Response	Microglia, Astrocytes, Inflammatory Factors	Injury and infection activate immune cells, release inflammatory mediators (e.g., TNF‐α, IL‐1β), affecting the excitability of nerve fibers, promoting pain signal generation.	
Central Nervous System (CNS)	CNS Feedback	Microglia, Astrocytes, Neurons, Descending Inhibitory Pathways, Endorphins	Glial cell activation releases inflammatory mediators, intensifying pain signals; sensitization of central neurons leads to an exaggerated pain response.	Central sensitization makes the spinal cord and brain more sensitive to pain signals from the periphery, leading to the amplification and persistence of pain sensation.

Glial cells, particularly microglia and astrocytes, play pivotal roles in neural‐immune regulation. They become activated in response to stimuli like nerve injury, inflammation, and pain‐related substances. Once activated, they release chemokines and inflammatory mediators, heightening activity in pain‐sensing neurons. This cascade prompts the synthesis and release of inflammatory factors, intensifying pain signal transmission.^26^, ^27^ The aggregation and activation of immune cells are also pivotal in pain sensitization, releasing inflammatory mediators at sites of nerve injury, which in turn induce neural sensitization and amplify pain perception.^28^

The interaction between the central and peripheral nervous systems is crucial in pain signal transmission. Central transmission can initiate pain perception, while sustained peripheral input exacerbates central nervous system sensitization.^41^ Moreover, the central nervous system regulates peripheral pain signal generation and processing via descending pathways.^38^ Bidirectional communication between the nervous and immune systems occurs, with the nervous system modulating immune cells through the release of neurotransmitters and neuropeptides, while immune cells influence the activity of nerve cells through released factors.^28^ This communication not only affects pain generation and sensitization but also alters pain perception.^1^

### 4.4 Neurodegenerative diseases, pain, and mental disorders: New perspectives and personalized treatments

The co‐occurrence network and keyword cluster analyses reveal intricate interconnections among neurodegenerative diseases, pain, and mental disorders. Over the past decade, investigations on Alzheimer's, chronic pain, ALS, and neuropathic pain have moved beyond causal inference toward clarifying the roles of inflammation, stress response, and oxidative stress in disease progression. Specifically, the Alzheimer's Disease cluster (#0) underscores the links between cognitive decline, chronic pain, and mental health issues such as depression and anxiety, suggesting that neurodegenerative processes may precipitate psychiatric symptoms and systemic immune disturbances, including chronic pain, which also act as risk factors for Alzheimer's disease through shared mechanisms like neuroinflammation and oxidative stress.^42^ The Amyotrophic Lateral Sclerosis cluster (#3) further illustrates how neurodegenerative progression in ALS leads to muscle and joint pain, which is compounded by anxiety and depression. Notably, emerging evidence points to a mechanistic link between chronic pain and neurodegenerative diseases, with persistent pain driving neuroinflammatory states and disruptions in protein homeostasis that parallel neurodegenerative processes.^43^ These advances reveal common biological pathways in pain generation and regulation, and they also drive the search for biomarkers and targeted interventions that enable more individualized care (Table 5).^44^

**Table 5:** Neurological and pain disorders: Mechanisms and responses.

Disease	Neurological damage and dysfunction	Activation of inflammatory response	Dysregulation of stress response	Increase in oxidative stress
Alzheimer's Disease	Abnormal accumulation of amyloid proteins; Pathological phosphorylation of tau protein	Activation of microglia and astrocytes, release of inflammatory mediators	Abnormal hormone levels exacerbating cognitive impairment	Oxidative stress accelerates neurodegenerative changes
Amyotrophic Lateral Sclerosis	Degeneration of motor neurons	Activation of microglia, release of inflammatory factors	Abnormal hormone levels accelerate neurodegeneration, endoplasmic reticulum stress (ER), abnormal protein folding	Oxidative stress leads to cellular damage
Chronic Pain	Chronic activation of the nervous system; Amplification of pain signals	Sensitization of pain pathways, continuous inflammation promotes the maintenance and exacerbation of pain	Stress hormone changes, intensifying pain perception	Oxidative stress causes damage to cellular components
Neuropathic Pain	Chronic activation of the nervous system; Amplification of pain signals	Enhanced pain perception, local inflammatory response, cytokine release	Hormonal level changes under stress	Excessive production of free radicals, cellular damage

Mechanistic studies demonstrate that Alzheimer's, chronic pain, ALS and neuropathic pain, despite distinct clinical manifestations, share features such as neuronal injury, persistent neuroinflammation, dysregulated hypothalamic–pituitary–adrenal (HPA) axis activity and elevated oxidative stress.^44^, ^45^, ^46^, ^47^, ^48^ These same processes are also implicated in psychiatric disorders. In Alzheimer's, inflammatory mediators affect neural circuits regulating mood and may lead to depression and anxiety.^46^ Parkinson's disease patients frequently experience depression and anxiety, possibly due to dopaminergic and serotonergic dysfunction.^45^ Chronic depression can disturb neuroendocrine homeostasis through HPA axis hyperactivity, thereby increasing vulnerability to neurodegeneration.^47^

Pain itself is a critical mediator of this bidirectional relationship. In neurodegenerative diseases such as Parkinson's disease or ALS, persistent nociceptive and neuropathic pain exacerbate psychological distress and contribute to depression and anxiety.^49^ Conversely, psychiatric disorders alter pain perception. Patients with depression or anxiety often display reduced pain thresholds and greater sensitivity, a phenomenon observed in chronic musculoskeletal pain where comorbidity with psychiatric disorders predicts poorer treatment outcomes.^50^ These reciprocal influences may create a vicious cycle that sustains both pain and mental symptoms.

Overall, the intersection of pain, neurodegeneration and psychiatric disorders highlights shared biological mechanisms that include inflammation, oxidative stress and stress‐response dysregulation.^44^, ^51^ Recognizing these overlaps provides a conceptual basis for cross‐disease therapeutic strategies. The rise of personalized medicine further enables treatments tailored to individual differences in pain perception, neuroimmune reactivity and psychiatric vulnerability, offering new opportunities to improve both symptom relief and quality of life. Future perspectives emphasize the development of personalized treatments that integrate neurological, immunological, and psychiatric dimensions. Biomarkers such as inflammatory cytokines, oxidative stress indicators, and genetic variations in the endocannabinoid system may help stratify patients according to their risk profiles. Interventions targeting microglial activation, oxidative stress pathways, or HPA axis regulation hold promise in simultaneously alleviating pain and psychiatric symptoms. Moreover, personalized medicine approaches—combining pharmacological treatments with psychological support and lifestyle interventions—can address individual variability in pain perception, neuroimmune reactivity, and psychiatric vulnerability, ultimately improving both symptom control and quality of life.

### 4.5 The complex relationship: Pain and mental health disorders

The relationship between pain and mental disorders is intricate and multifaceted (Table 6). Studies have consistently shown that chronic pain often coincides with depressive symptoms, and individuals with depression frequently report experiencing various physical pains.^52^ This overlap is partly explained by shared neurochemical mechanisms, including dysfunction in the HPA axis, as well as abnormalities in serotonin and noradrenaline pathways.^38^, ^53^, ^54^

**Table 6:** Interrelationship between pain and mental disorders: Mechanisms, manifestations, and treatment considerations.

Direction of influence	Pain → Mental disorders	Mental disorders → Pain
Common Neurochemical Mechanisms	Dysfunction of the Hypothalamic‐Pituitary‐Adrenal (HPA) axis, abnormalities in serotonin and norepinephrine pathways	
Specific Manifestations	Chronic pain accompanied by symptoms such as anxiety, depression, and sleep disturbances	Patients with mental disorders experience more frequent, more intense, and longer‐lasting pain
Neurochemical Mechanisms	Dysfunction of the HPA axis, abnormalities in serotonin and norepinephrine pathways	Changes in attention and pain processing areas (e.g., anterior cingulate cortex)
Biological Pathways	Common inflammatory response, changes in neurotransmitters	Long‐term changes in brain structure and function (neuroplasticity)
Behavioral and Cognitive Impacts	Chronic pain can lead to cognitive impairment, such as decreased attention	Emotional issues intensify the pain experience, leading to attentional bias towards pain information, decreased attention
Treatment Differences	Traditional analgesics are less effective for pain caused by depression	Treatment of depression may need to be combined with pain management

Both tissue damage and depression can lead to heightened pain sensitivity. However, traditional analgesics often demonstrate limited efficacy in treating depression‐related pain, suggesting differing mechanisms underlying pain generation.^55^ Animal studies have demonstrated that pain resulting from tissue damage operates through distinct neural pathways compared to pain triggered by depressive states.^55^ Furthermore, pain and mental disorders may trigger common biological responses, such as inflammation and changes in neurotransmitters, which can influence individuals' perception of pain and emotional well‐being. Additionally, chronic pain has been linked to cognitive impairment, including alterations in attention and the interaction between pain perception and cognition.^56^ Studies have indicated that individuals with chronic pain often exhibit a heightened focus on pain‐related information, which significantly contributes to their cognitive dysfunction.^57^, ^58^

Clinical observations further support the notion that chronic pain frequently co‐occurs with symptoms of depression and anxiety, with emotional issues exacerbating the frequency and intensity of pain episodes.^59^, ^60^ Negative emotions not only activate brain regions associated with pain but may also exacerbate patients' perception of pain.^61^ Research suggests that depression has different effects on various types of pain, such as attenuating pain induced by transient stimuli but enhancing spontaneous pain.^62^

### 4.6 Deep cross‐disciplinary research: Frontiers of pain and mental disorders

Cutting‐edge interdisciplinary research is advancing through the fusion of various fields, particularly in understanding complex biological phenomena like pain, mental disorders, inflammation, and oxidative stress. By integrating insights from neuroscience, psychology, molecular biology, and genetics, scientists are unraveling the biological roots of pain while acknowledging its diverse and subjective nature. This approach not only drives the development of innovative treatments, such as psychological interventions for pain management but also sheds light on the intricate interplay between stress, psychology, and immune function, underscoring the significance of psychological strategies in mitigating stress‐related health issues and autoimmune diseases. Moreover, the convergence of disciplines like cell biology, biochemistry, nutrition, and metabolism is unveiling strategies to modulate inflammation and oxidative stress through dietary interventions.^63^ Leveraging bioinformatics, computational biology, and advanced imaging techniques further enhances our understanding of these complex processes at molecular and systemic levels, facilitating early disease diagnosis and treatment.^64^

### 4.7 Advantages and limitations

This study provides a thorough examination of the intricate relationship between pain and mental disorders, along with their underlying neuroimmune mechanisms, drawing from a comprehensive review of literature. Its interdisciplinary approach allows for nuanced insights and uncovers new research avenues.

However, this study has several limitations. First, while we focused on PubMed, Web of Science, and Scopus to ensure high‐quality and comprehensive literature inclusion, relevant studies from other sources may have been missed. Future research could address this by expanding the database scope and using interdisciplinary databases, along with sensitivity analyses to enhance reliability. Second, our bibliometric analysis is limited to a specific time period, which excludes studies published after the initial search. This time gap, inherent to bibliometric analysis, minimally impacts the trends within the defined timeframe but could be mitigated in future studies by updating the data. Lastly, variations in author names, institution names, and keywords caused some dispersion in counts and clustering. We addressed this through manual correction and normalization during data cleaning to minimize inconsistencies.

## 5 CONCLUSION

This study uses bibliometric analysis to explore the complex relationship between pain and mental disorders, emphasizing neuroimmune mechanisms and the importance of interdisciplinary research. Over the past decade, there has been sustained interest in the intersection of neurodegenerative diseases, pain disorders, and mental illness. Future research should integrate the biopsychosocial model with neuroimaging, genomics, and molecular biology to identify biomarkers and develop tools for early diagnosis and precision treatment. Clinical trials across diverse populations are also needed to validate personalized therapies and assess the impact of social and environmental factors. Strengthening interdisciplinary and international collaboration will further advance the field, leading to more precise and individualized treatment options for patients.

## AUTHOR CONTRIBUTIONS

Zhimin Tan designed the study, performed data analysis, and wrote and drafted the article. Qiyu He contributed to writing and drafting the article. Xian Zhang critically revised the article. Jiarong Feng provided software operation guidance and edited the manuscript. Yuxin He conducted data analysis. Xiaoqiang Li critically revised and approved the final version of the article.

## CONFLICT OF INTEREST STATEMENT

The authors declare no conflicts of interest.

## ETHICS STATEMENT

The ethics statement is not available.

## Data Availability

The data generated and/or analyzed during the current study are available from the corresponding author upon reasonable request.
